# Rationality assessment of postoperative disc-condyle position following TMJ arthroscopic disc repositioning operation: a retrospective MRI study

**DOI:** 10.1186/s12903-026-08275-w

**Published:** 2026-06-11

**Authors:** Chenxi Jin, Na Li, Jianfeng Sun, Wei Shen, Dongyu Hou, Chi Yang, Jing Guo

**Affiliations:** 1 Ningbo Stomatology Hospital, Ningbo, China; 2 School of Basic Medical Sciences and Forensic Medicine, Hangzhou Medical College, Hangzhou, China; 3 Department of Oral Surgery, Ninth People’s Hospital, Shanghai Jiao Tong University School of Medicine, Shanghai Key Laboratory of Stomatology and Shanghai Research Institute of Stomatology, National Clinical Research Center of Stomatology, Shanghai, China

**Keywords:** Temporomandibular joint, Temporomandibular joint disorders, Arthroscopy, Magnetic resonance imaging, Range of motion

## Abstract

**Objectives:**

To systematically evaluate the postoperative disc-condyle relationship—including both structural position and functional mobility—following bilateral arthroscopic disc repositioning (Bi-ADRO) for temporomandibular joint disorders.

**Material and methods:**

This retrospective study initially enrolled 90 consecutive patients who underwent MRI. After applying predefined inclusion and exclusion criteria, 31 patients without anterior disc displacement (non-ADD) were assigned to the symptomatic control group (group N), and 59 patients (101 joints) diagnosed with anterior disc displacement without reduction (ADDWoR) treated by Bi-ADRO constituted the surgical group (group A). Postoperative MRI was performed at 1, 3, and 6 months (A_1M_ ~ A_6M_). Statistical analyses were conducted using IBM SPSS Statistics v.27.0, with *p* < 0.05 considered statistically significant.

**Results:**

Preoperatively, group A_0_ showed significantly lower maximum mouth opening (MMO) and greater disc-condyle angular deviations than group N ( *p* < 0.001). After Bi-ADRO, MMO improved progressively at all follow-ups ( *p* < 0.05). Sagittal disc position improved markedly in A_1M_ ~ A_6M_, but coronal alignment did not change significantly. Disc mobility increased most markedly by the third month, with vertical motion exceeding horizontal motion, whereas condylar mobility remained predominantly horizontal. At 6 months, 51.5% of joints achieved a disc-condyle angle between 0° and -60°. Changes in disc-condyle angle from 1 to 6 months were negatively correlated with the surgical disc position (A_1M~3 M_: *p* < 0.001, *r* = -0.457; A_1M~6 M_: *p* < 0.001, *r* = -0.499).

**Conclusion:**

Bi-ADRO restores sagittal disc position and improves joint mobility in patients with ADDWoR, although coronal disc displacement may persist. The degree of surgical overcorrection influences postoperative disc stability and coordinated disc-condylar movement. These findings support the use of quantitative MRI-based angular targets to optimize surgical outcomes, though longer-term studies are needed to confirm durability.

## Introduction

The temporomandibular joint (TMJ) is a complex structure essential for mastication and speech, comprising the glenoid fossa, articular eminence, condyle, articular disc, and associated ligaments [1–3]. Its anatomical and biomechanical complexity renders it susceptible to pathological changes [4]. Temporomandibular disorders (TMD) are prevalent, with reported rates of 45—54% in various populations [5–7]. Anterior disc displacement (ADD) represents the most common TMD subtype, affecting 18—25% of individuals and characterized by an abnormal articular disc position [8, 9].

ADD is particularly concerning in adolescents, where it may inhibit mandibular growth and contribute to dentofacial deformities [10–13]. Schellhas et al. [14] reported that 112 of 128 orthodontic patients under 14 years presented with ADD, and among those with mandibular retrusion, 93% had bilateral ADD. In skeletal Class II malocclusion, disc displacement prevalence reaches 78%, significantly exceeding that in Class I (61%) and Class III (67%) [15]. These associations underscore the importance of early diagnosis and intervention to prevent progressive joint degeneration and facilitate normal maxillofacial development.

Treatment modalities for ADD range from conservative management to surgical intervention. Arthroscopic disc repositioning operations (ADRO), introduced by Tarro in 1989 [16] and refined by subsequent investigators [17–23], have demonstrated success in restoring disc position and improving joint symptoms. Yang's procedure, in particular, achieved successful sagittal disc repositioning in 98.6% of 764 joints [22]. In adolescents, ADRO may restore condylar growth potential by normalizing intra-articular biomechanics [24–28]. However, excessive surgical overcorrection may generate undue posterior attachment tension, potentially limiting disc mobility and long-term adaptability.

Previous evaluations of surgical outcomes have relied primarily on CT imaging, which cannot visualize the disc and involves ionizing radiation [29, 30]. Magnetic resonance imaging (MRI) offers superior soft-tissue resolution and is considered the gold standard for assessing disc position and morphology [31, 32]. While most studies focus on sagittal plane analysis, the TMJ is a three-dimensional structure, and up to one-third of anteriorly displaced discs also exhibit coronal displacement [33]. Failure to evaluate coronal images may result in 13% false-negative diagnoses [34–37], underscoring the need for comprehensive biplanar assessment.

Despite advances in surgical technique, most prior studies have been limited to static structural descriptions, lacked appropriate control groups, and failed to assess dynamic functional outcomes. The relationship between the degree of surgical overcorrection and postoperative disc-condyle mobility remains poorly understood, and evidence-based guidelines for optimal surgical positioning are lacking.

Therefore, this study aimed to evaluate the postoperative disc-condyle relationship—encompassing both structural position and functional mobility—following bilateral ADRO (Bi-ADRO) in patients with ADD without reduction (ADDWoR). Using serial sagittal and coronal MRI at 1, 3, and 6 months postoperatively, we quantitatively analyzed disc-condyle angles and mobility patterns. The null hypothesis was that no significant correlation exists between the surgical disc position and postoperative changes in disc-condyle angle or mobility.

## Materials and methods

### Study design

**Fig. 1:**
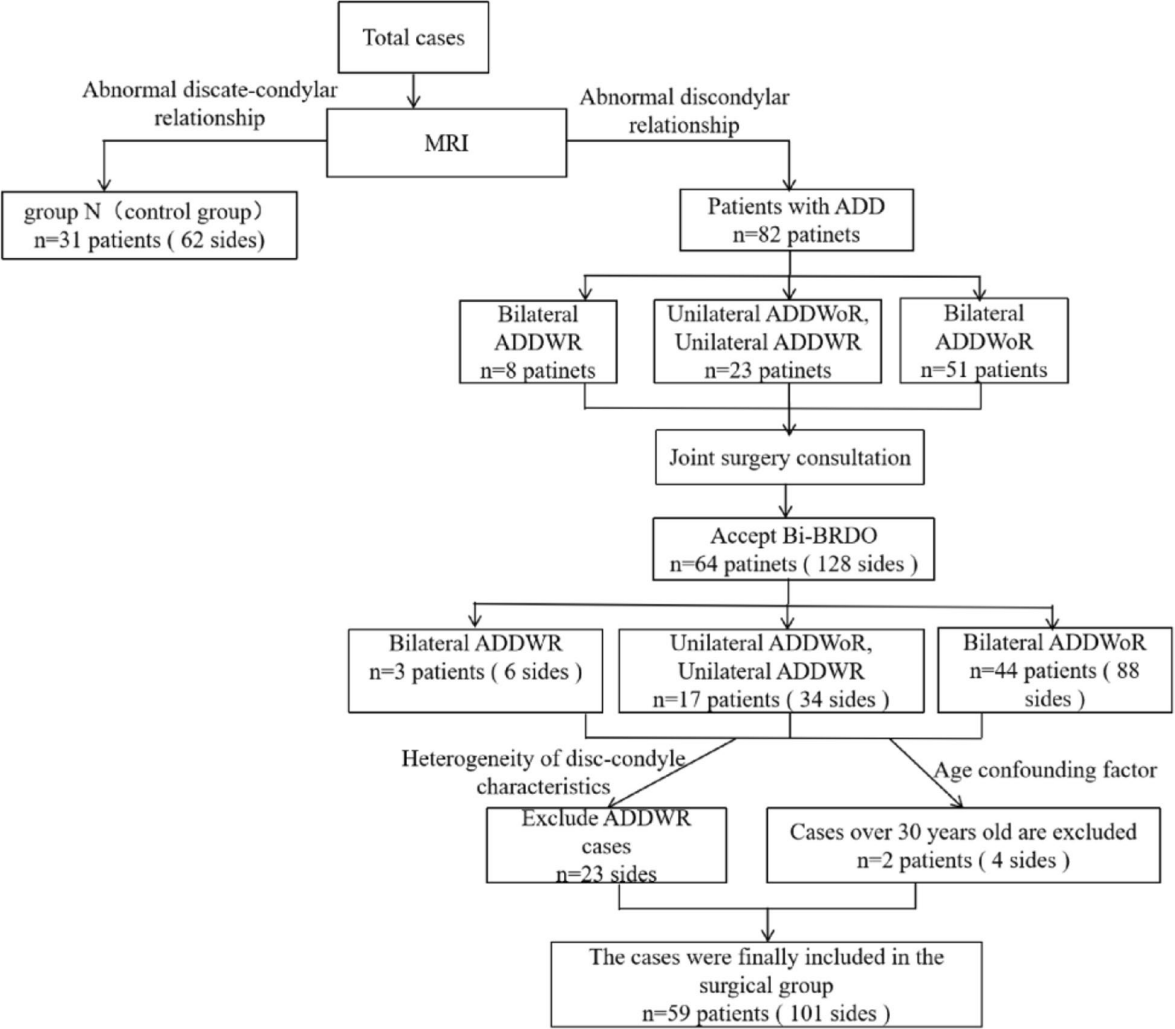
The screening process for the control group and the surgical group

A total of 90 consecutive patients presenting with myalgia or temporomandibular joint dysfunction as the primary complaint, who attended Ningbo Stomatology Hospital between January 2021 and February 2025, were initially enrolled. After applying the predefined inclusion and exclusion criteria, 31 patients were assigned to the non-ADD symptomatic control group (group N), and 59 patients (101 joints) diagnosed with anterior disc displacement without reduction (ADDWoR) who underwent bilateral arthroscopic disc repositioning (Bi-ADRO) constituted the surgical group (group A). The screening process is detailed in Fig. 1. This study was approved by the Ethics Committee of Ningbo Stomatology Hospital (Approval No. NBKQYY2024LS-027) on July 29, 2024, and was conducted in accordance with the principles of the Declaration of Helsinki. Written informed consent was obtained from all participants. For minors under 18 years of age, informed consent was obtained from their parents or legal guardians.

Inclusion criteria: (1) patients presenting with mandibular retrusion or deviation, myalgia, or TMD who underwent MRI; (2) all patients underwent Bi-ADRO performed by the same surgeon; (3) patients who completed clinical follow-ups at 1, 3, and 6 months postoperatively with MRI.

Exclusion criteria: (1) severe medial or lateral rotational disc displacement; (2) history of joint infection, jaw fracture, or congenital/systemic diseases potentially affecting jaw development; (3) poor-quality MRI or CT images; (4) orthognathic surgery during the observation period; (5) significant psychosomatic disorders; (6) previous arthroplasty; (7) incomplete medical information.

The control cohort (non-ADD symptomatic control group, group N) comprised patients presenting with facial myalgia or TMJ discomfort but diagnosed via MRI as having no anterior disc displacement. This group was included not as an asymptomatic ideal, but as a clinically relevant comparator representing patients seeking care for TMJ-related symptoms, against which structural abnormalities in the ADD group could be contrasted.

### Surgical techniques

#### Arthroscopic technique

All surgeries were performed under local or general anesthesia by the same experienced surgeon. A temporomandibular arthroscopic triple trocar puncture technique was used to access the superior joint space. Release of the anterior disc attachment was accomplished under direct arthroscopic visualization via puncture through the anterior synovial fold. Using an arthroscopic suture needle (2—0 PDS II; Ethicon, Somerville, NJ, USA), the superior joint space was punctured to engage the disc at the junction of its posterior band and the bilaminar zone. The needle was passed from the junction of the middle and outer thirds of the disc. A suture needle with a thread retriever (Smith & Nephew, London, UK) was then introduced through an external auditory canal incision to hook and extract the suture from the joint cavity. This process was repeated twice to complete a horizontal mattress suture fixation of the disc using a two-needle double-suture technique [38].

#### MRI acquisition

All TMJ MRI examinations were conducted by the same radiologist in the Department of Radiology, Ningbo Stomatology Hospital, preoperatively and at 1, 3, and 6 months postoperatively. Imaging was performed using a 1.5-T MRI system (uMR 586; Shanghai Lianxiang Medical Technology, Shanghai, China) with a dedicated head coil.

Patients were positioned supine with the head stabilized and aligned such that the sagittal and orbitomeatal planes were perpendicular to the scanner table. Surface coils were centered on the external auditory canal and placed as close as possible to both TMJ regions. The open-mouth position was standardized as the maximum comfortable, pain-free opening that the patient could actively achieve and maintain. This position was verbally instructed and visually confirmed by the attending radiologist immediately before and during scanning to ensure consistency and minimize pain-related muscular resistance or guarding.

The following scan sequences were acquired:

- T1-weighted (T1W): Repetition time (TR) 498 ms, echo time (TE) 11.04 ms, field of view (FOV) 13 cm, slice thickness 3.0 mm. Scanning planes: transverse T1W in closed position to determine the bilateral condylar long-axis line; sagittal and coronal T1W images perpendicular and parallel to this line, respectively, including sagittal closed, sagittal open, and coronal closed positions.
- T2-weighted (T2W): TR 3200 ms, TE 54.6 ms, FOV 13 cm, slice thickness 3.0 mm. Scanning plane: sagittal open-mouth T2W.

#### MRI evaluation

All preoperative and postoperative MRI images (1, 3, and 6 months) were reviewed and measured by the same experienced orthodontist. Measurements were performed three times by the same observer. After a two-week interval, 30 joint samples were randomly selected for re-measurement. Intra-observer agreement was assessed using the intraclass correlation coefficient (ICC).

#### MRI measurements

Oblique sagittal MRI images showing the maximum cross-section of the articular disc in both closed and open-mouth positions were selected, transferred to a computer, and reference points were determined for measurement.

#### Articular disc-condyle angle

**Fig. 2:**
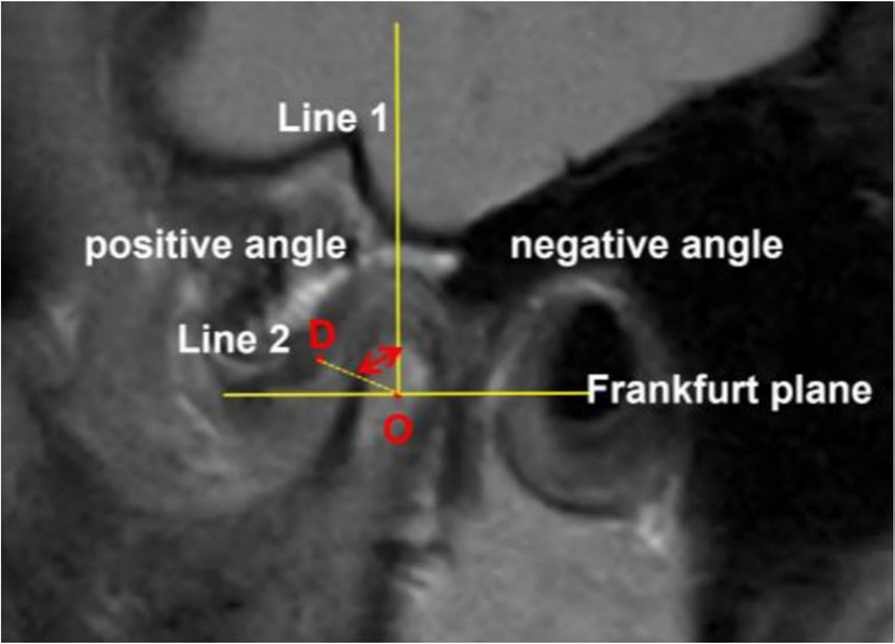
Measurement of disc-condyle angle using the Drace-Enzmann method. Point O: condylar center; Point D: midpoint of the posterior band of the disc. The angle between line OD and the plump line (perpendicular to the Frankfurt plane) defines the disc-condyle angle

The Drace-Enzmann method [39] was used. Point O was defined as the condylar center and point D as the midpoint of the posterior margin of the disc's posterior band. A plumb line perpendicular to the Frankfurt plane was drawn through point O. The angle between the line OD and this plumb line was defined as the disc-condyle angle (Fig. 2). Based on the condylar center as a reference, disc positions were categorized as follows: Advantageous overcorrection (angle between 0° to −60°); True overcorrection (angle exceeding −60°); Splint-dependent (angle between 0° to 90°); and Relapse (angle exceeding 90°).

#### Measurement of articular disc and condyle position [40]

**Fig. 3:**
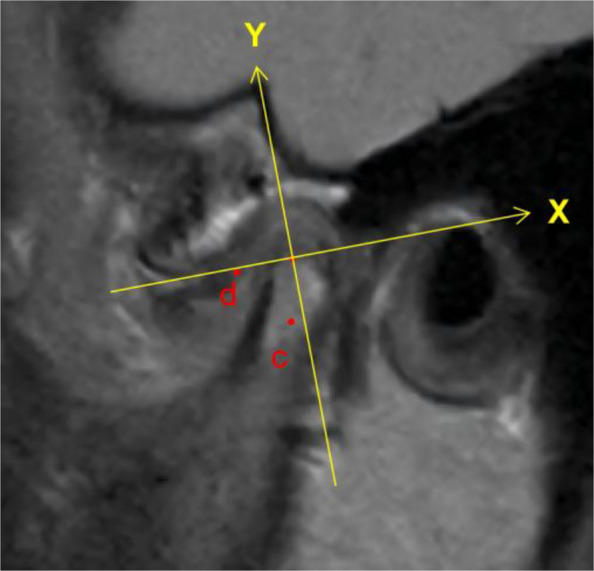
Coordinate system for measuring disc and condyle position. X- axis: line connecting the lowest point of the articular eminence and the most convex point of the external auditory canal. Y-axis: perpendicular to X-axis through the highest point of the glenoid fossa. Origin (O): intersection of X and Y axes. Poins c (condyle center) and d (midpoint of disc posterior band) are plotted

A coordinate measurement method was employed (Fig. 3). The X-axis was defined as the line connecting the lowest point of the articular eminence and the most convex point of the external auditory canal. The Y-axis was defined as the perpendicular to the X-axis passing through the highest point of the glenoid fossa, with the intersection designated as point O (the origin). Points c (condyle center) and d (midpoint of the disc's posterior band) were plotted. Positional changes of the disc and condyle were analyzed based on changes in the (x, y) coordinates of points c and d, relative to the quadrants defined by the origin.

#### Medial–lateral disc displacement

**Fig. 4:**
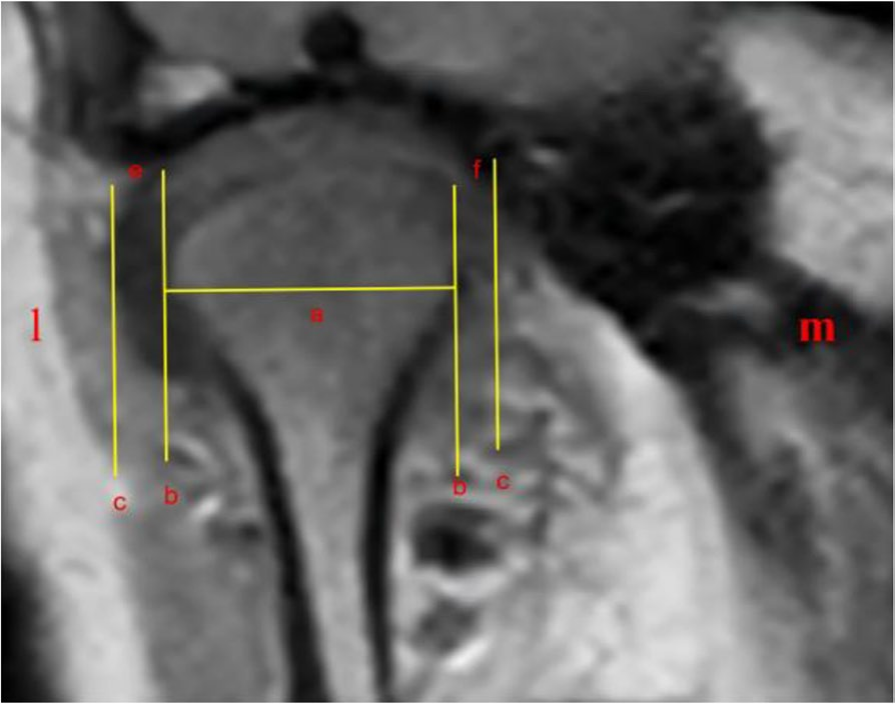
Schematic diagram illustrating the measurement of media-lateral displacement of the articular disc (right temporomandibular joint, coronal view). Line a represents the maximum mediolateral diameter of the mandibular condyle. Line b is drawn perpendicular to line a at the inner and outer edges of the articular disc. The displacement is defined by the distances e (lateral displacement) and f (medical displacement) l: material; m: medial

The method described by Schmitter et al. [41] was used. In the normal coronal position, the disc is centered over the condyle. Medial or lateral displacement was identified when the disc expanded at one end and shifted towards that side relative to the condylar margins (Fig. 4).

### Statistical analysis

Statistical analyses were performed using IBM SPSS Statistics (version 27.0; IBM, Armonk, NY, USA), with *p* < 0.05 considered statistically significant. Due to the retrospective nature of the study, a priori sample size calculation was not conducted. To minimize selection bias, all consecutive patients who met the eligibility criteria during the study period were included. The final sample comprised 90 patients, including 101 joints in group A and 62 joints in group N, determined by the availability of complete data over the recruitment period. Post-hoc power analysis was not performed; instead, effect sizes and confidence intervals are reported to aid in the interpretation of findings and to inform future prospective studies. All MRI measurements were performed by a single experienced orthodontist to ensure consistency, and intra-observer reliability was excellent (ICC > 0.90). Normality of the data was assessed using the Shapiro–Wilk test. The Mann–Whitney U test was used to compare disc-condyle angle and mobility between groups. One-way analysis of variance (ANOVA) was performed to evaluate changes in these parameters at different time points within group A, followed by post-hoc least significant difference (LSD) tests for pairwise comparisons. Pearson correlation analysis was used to examine the relationship between surgical disc positioning angles and changes in disc-condyle mobility.

## Results

### Sample characteristics

The demographic and clinical characteristics of the study population are summarized as follows: The non-ADD symptomatic control group (group N) comprised 31 patients (62 joints) with a median age of 18.0 years (range: 12–45 years), including 24 females (77.4%) and 7 males (22.6%). The surgical group (group A) included 59 patients (101 joints) with a median age of 16.0 years (range: 11–38 years), consisting of 48 females (81.4%) and 11 males (18.6%). The predominance of female patients in both groups is consistent with the known epidemiology of TMD. The slightly younger median age in group A reflects the growing recognition of ADD in adolescent populations and the importance of early surgical intervention. Complete follow-up at 1, 3, and 6 months postoperatively was achieved for all 59 surgical patients.

### Changes in Maximum Mouth Opening (MMO)

Table 1 presents the changes in pain-free maximum mouth opening across all study groups and time points. Preoperatively, group A_0_ demonstrated significantly lower MMO (32.80 ± 11.21 mm) compared to group N (46.23 ± 4.86 mm; *p* < 0.001), confirming the functional limitation associated with ADDWoR.

**Table 1:** Comparison of MMO among groups and different time points (mean ± SD)

Measurement	group N		group A_0_	group A_1M_	group A_3M_	group A_6M_
	initial diagnosis	Physical therapy/observation and follow-up				
MMO(mm)	46.23 ± 4.86*	48.42 ± 3.93	32.80 ± 11.21	32.97 ± 4.34a	40.86 ± 5.68ab	44.92 ± 5.04abc
*p*-value	< 0.05					

a vs. group A_0_, *p* < 0.05; b vs. group A_1M_, *p* < 0.05; c vs. group A_3M_, *p* < 0.05. All pairwise comparisons were adjusted by the LSD test

^*^vs. group A_0_, *p* < 0.05

Following Bi-ADRO, progressive improvement in MMO was observed at all postoperative intervals. At 1 month postoperatively (A_1M_), mean MMO increased to 32.97 ± 4.34 mm (*p* < 0.05 vs. A_0_), further improving to 40.86 ± 5.68 mm at 3 months (A_3M_; *p* < 0.05 vs. A_1M_) and reaching 44.92 ± 5.04 mm at 6 months (A_6M_; *p* < 0.05 vs. A_3M_). Although A_6M_ values approached those of group N, the difference remained statistically significant (*p* < 0.05), indicating that functional recovery, while substantial, was not complete within the 6-month follow-up period.

### Preoperative comparison of disc-condyle angle and mobility: group N vs. Group A_0_

**Fig. 5:**
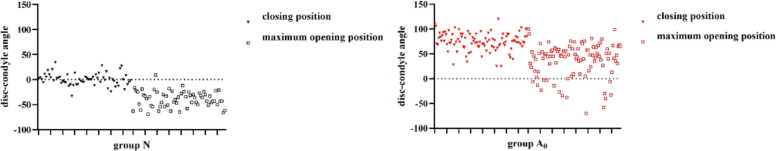
Comparison of preoperative disc-condyle angles between Group N (asymptomatic volunteers) and Group A_0_ (patients with anterior disc displacement without reduction). The box-and-whisker plots show the angles measured at the closing position (left) and the maximum opening position (right). The dashed line indicates the reference angle 0. Positive values indicate anterior displacement of the disc relative to the condyle

**Fig. 6:**
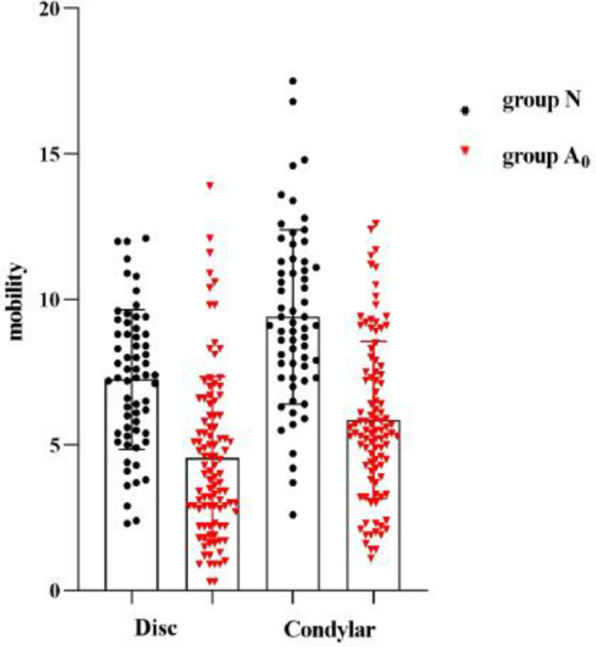
Comparison of mobility between group N and group A_0_

Table 2 and Figs. 5 and 6 summarize the comparative analysis of disc-condyle angles and mobility between the non-ADD control group and the preoperative surgical group.

**Table 2:** Comparison of TMJ MRI measurements among groups and different time points (mean ± SD)

Measurement	N	A_0_	A_1M_	A_3M_	A_6M_	*p*-value
Disc-condyle angle (closed)	0.39° ± 12.22°	74.61° ± 18.04°	−72.27° ± 29.80°a	−61.46° ± 31.35°ab	−57.98° ± 31.76°ab	< 0.001
Disc-condyle angle (open)	−38.79° ± 15.72°	37.45° ± 34.00°	−72.04° ± 33.72°a	−53.19° ± 25.53°ab	−49.88° ± 24.83°ab	< 0.001
Disc mobility (mm)	7.248 ± 2.398	4.545 ± 2.802	3.254 ± 2.615a	3.136 ± 2.123a	3.262 ± 2.225a	< 0.001
Condyle mobility (mm)	9.395 ± 2.991	5.853 ± 2.701	1.758 ± 1.597a	2.995 ± 2.303ab	3.380 ± 2.438ab	< 0.001
Lateral displacement (sides)	3(4.84%)	0(0)	1(4.34%)	2(8.33%)	1(2.94%)	-
Medial displacement (sides)	3(4.84%)	0(0)	5(21.74%)	3(12.5%)	9(26.47%)	-
Visible disc (sides)	62(100%)	0(0)	23(23.23%)	24(24.24%)	34(34.34%)	-

a vs. group A_0_, *p* < 0.05; b vs. group A_1M_, *p* < 0.05; c vs. group A_3M_, *p* < 0.05. All pairwise comparisons were adjusted by the LSD test

In the coronal plane, lateral disc displacement was observed in only 9.68% of joints in group N, whereas discs were rarely discernible in group A_0_ due to their displaced position, confirming the three-dimensional nature of ADD pathology.

In the sagittal plane, significant differences in disc-condyle angles were observed between groups in both closed and open positions. At closed mouth, group N exhibited a mean disc-condyle angle of 0.39° ± 12.22°, while group A_0_ showed a markedly displaced angle of 74.61° ± 18.04° (*p* < 0.001). At maximum opening, group N demonstrated physiological disc translation with a mean angle of –38.79° ± 15.72°, whereas group A_0_ showed minimal angular change (37.45° ± 34.00°; *p* < 0.001), reflecting the characteristic locking and restricted disc mobility in ADDWoR.

Disc-condyle mobility during opening–closing movements, calculated as the difference between open and closed positions, was significantly reduced in group A_0_ (disc: 4.55 ± 2.80 mm; condyle: 5.85 ± 2.70 mm) compared to group N (disc: 7.25 ± 2.40 mm; condyle: 9.40 ± 2.91 mm; both *p* < 0.001). These findings confirm that pathological disc-condyle relationships impair functional joint kinematics and establish the baseline abnormalities against which postoperative improvements were measured.

### Postoperative changes in disc-condyle angle and mobility over time

As shown in Table 2, progressive changes in disc-condyle angle and mobility were observed over the 6-month follow-up period.

In the coronal view, articular discs were visualized in 23, 47, and 81 joints in groups A_1M_, A_3M_ and A_6M_, respectively. Lateral disc displacement was present in 6/23 (26.1%), 5/24 (20.8%), and 10/34 (29.4%) visualized joints at 1, 3, and 6 months, respectively, indicating persistent coronal instability despite sagittal correction.

In the sagittal view, disc-condyle angles progressively increased over time, though the rate of increase slowed after 3 months. Significant differences were found between group A_1M_ and A_3M_ (*p* < 0.05) and between A_1M_ and A_6M_ (*p* < 0.05), but not between A_3M_ and A_6M_ (*p* > 0.05), suggesting stabilization of disc position after the third month.

Disc-condyle mobility showed continuous improvement from A_1M_ to A_6M_, with significant differences between each consecutive time point (*p* < 0.05). Motion pattern analysis revealed that disc movement was initially more vertical (A_1M~_A_3M_), becoming more sagittal thereafter (A_3M~_A_6M_), while condylar motion remained predominantly sagittal throughout.

### Comparison of disc-condyle angle and mobility between group A6M and N

Results at 6 months are summarized in Table 2. In the coronal view, discs were visible in 81 of 99 joints (81.8%) in group A_6M_, with lateral displacement observed in 21 of 99 visualized joints. In contrast, discs were consistently visible in all group N joints, with minimal lateral displacement (9.68%).

In the sagittal view, significant differences in disc-condyle angles persisted between group A_6M_ and group N in both closed and open positions (*p* < 0.001). Notably, 51 of 99 joints (51.5%) in group A_6M_ exhibited disc-condyle angles within the range of 0° to −60°, defined as “advantageous overcorrection”.

Disc-condyle mobility remained significantly lower in group A_6M_ compared to group N (*p* < 0.001), indicating that while substantial functional improvement occurred, complete biomechanical normalization was not achieved within 6 months.

### Correlation between postoperative disc-condyle mobility and MMO

We performed a formal Pearson correlation analysis using individual patient data from the surgical group (group A) at each postoperative time point. The results are as follows: at 1 month, disc mobility showed a weak, non‑significant correlation with MMO (*r* = 0.21, *p* > 0.05), and condyle mobility also showed a weak correlation (*r* = 0.18, *p* > 0.05). At 3 months, both parameters demonstrated modest but statistically significant positive correlations with MMO (disc: *r* = 0.34, *p* < 0.05; condyle: *r* = 0.29, *p* < 0.05). At 6 months, the correlations remained significant (disc: *r* = 0.28, *p* < 0.05; condyle: *r* = 0.31, *p* < 0.05).

### Postoperative mobility of articular disc and condyle relative to surgical disc position

Table 3 presents postoperative disc and condyle mobility at 1, 3, and 6 months, stratified by intraoperative surgical disc position angle (categorized as 0° to −30°, −30° to −60°, and −60° to −90°). Joints with surgical angles within 0° to −30° demonstrated the greatest improvement in both disc and condylar mobility, whereas those with angles exceeding −60° showed limited disc mobility improvement despite progressive increase in condylar mobility over time.

**Table 3:** Postoperative disc-condyle mobility and 6-month angular outcomes stratified by surgical disc repositioning angle (mean ± SD)

Surgical Position Angle of Articular Disc	Condyle Mobility(mm)			Disc Mobility(mm)			disc-condyle angle(closed position)	disc-condyle angle(maximum opening position)
	A_1M_	A_3M_	A_6M_	A_1M_	A_3M_	A_6M_		
0° ~ −30°	3.614 ± 3.143	4.529 ± 5.063	3.914 ± 5.082	4.243 ± 3.611	4.000 ± 4.083	3.329 ± 4.140	−11.86° ± 26.74°	−31.57° ± 29.66°
−30° ~ −60°	2.131 ± 1.896	3.090 ± 2.500	3.597 ± 2.315	3.448 ± 2.374	3.076 ± 2.030	3.507 ± 1.774	−49.55° ± 35.81°	−39.52° ± 27.02°
−60° ~ −90°	1.420 ± 0.844	2.800 ± 1.815	3.229 ± 2.049	2.736 ± 2.041	3.029 ± 1.762	3.373 ± 2.314	−63.87° ± 24.46°	−54.69° ± 20.68°

### Correlation between Bi-ADRO surgical disc position and disc-condyle mobility

**Fig. 7:**
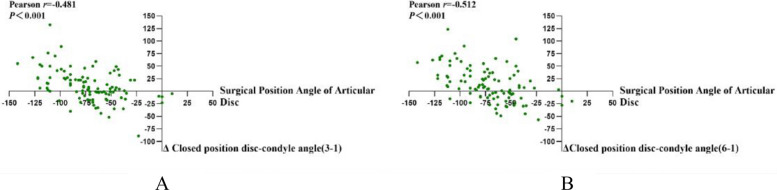
Analysis of the correlation between the changes of disc-condyle angle from the first to the third and the third and to sixth month **A** Correlation between the changes of disc-condyle angle from the first to the third month postperatively and first month postperatively; **B** Correlation between the changes of disc-condyle angle from the first to the sixth month postperatively and first month postperatively

As presented in Table 4 and Fig. 7, temporal changes in the disc-condyle angle from 1 to 3 months and from 1 to 6 months were negatively correlated with the surgical disc position angle at closed mouth (1—3 months: *r* = –0.457, *p* < 0.001; 1—6 months: *r* = –0.499, *p* < 0.001). This indicates that a more posterior surgical disc position (more negative angle) was associated with less angular change over time, suggesting greater postoperative stability.

**Table 4:** Correlation between Bi-ADRO surgical disc position and disc-condyle mobility

measurement parameter		Δ Closed position disc-condyle angle(3—1)	Δ Closed position disc-condyle angle(6—1)	Δ Open position disc-condyle angle (3—1)	Δ Open position disc-condyle angle (6—1)
Surgical Position Angle of Articular Disc	*r*	−0.481**	−0.512**	−0.191	−0.160
	*p*	< 0.001	< 0.001	0.056	0.109

^*^p < 0.05, **p < 0.01

Table 3 provides detailed 6-month postoperative disc-condyle angles stratified by surgical position category. In the 0° to −30° group, mean closed-mouth angle was −13.33° ± 9.79° and open-mouth angle was −24.83° ± 25.96°. In the −30° to −60° group, corresponding values were −49.55° ± 35.81° (closed) and −39.52° ± 27.02° (open). In the −60° to −90° group, angles measured −63.27° ± 24.41° (closed) and −54.23° ± 20.68° (open).

## Discussion

This study aimed to test the null hypothesis that no significant correlation exists between surgical disc position and postoperative changes in disc-condyle mobility. Our findings reject this null hypothesis, demonstrating significant negative correlations at both 3-month (*r* = −0.457, *p* < 0.001) and 6-month (*r* = −0.499, *p* < 0.001) follow-ups. These results confirm that intraoperative disc positioning influences postoperative joint dynamics and support the clinical utility of quantitative angular targets in surgical planning.

### Surgical outcomes and optimal disc position

Consistent with previous studies [17–23], Bi-ADRO significantly improved sagittal disc position and maximum mouth opening (MMO) in patients with ADDWoR. The progressive improvement in MMO from 1 to 6 months postoperatively indicates functional recovery, although values remained slightly below those of the non-ADD control group at 6 months, suggesting that complete normalization of joint function may require more time. Notably, 51.5% of joints achieved a disc-condyle angle within the range of 0° to −60° at 6 months, which we defined as “advantageous overcorrection”. Joints within this range exhibited greater improvements in disc and condylar mobility, as well as stronger negative correlations with favorable temporal changes in disc-condyle angle, indicating greater postoperative stability. These findings align with previous reports emphasizing the importance of precise disc positioning [22, 42] and extend them by providing a quantifiable target based on longitudinal kinematic data.

In contrast, angles exceeding −60° (“true overcorrection”) were associated with limited disc mobility improvement, possibly due to increased tension in the posterior attachment [43, 44]. This suggests that excessive overcorrection may restrict elastic deformation of the bilaminar zone and hinder coordinated disc-condyle movement. Conversely, angles between 0° and 90° (“splint-dependent”) or exceeding 90° (“relapse”) reflect inadequate correction or postoperative instability, underscoring the need for individualized surgical planning.

### Persistent coronal displacement

Despite marked sagittal improvement, coronal disc displacement did not change significantly after Bi-ADRO. This finding is consistent with previous observations that up to one-third of anteriorly displaced discs also exhibit coronal displacement [33, 36], and that sagittal repositioning alone may not fully address mediolateral instability. The persistence of lateral displacement in 10 of 34 visualized joints at 6 months suggests that current arthroscopic techniques, while effective in the sagittal plane, have inherent limitations in controlling coronal alignment. This may be due to the surgical focus on anterior–posterior correction, less direct addressing of mediolateral stabilizing structures by standard suturing, or the rigidity of pre-existing coronal deformities. Future refinements in surgical technique, such as adjunctive coronal stabilization maneuvers, may be necessary to achieve three-dimensional disc reduction.

### Biological heterogeneity and confounding variables

An important limitation of this study is the biological heterogeneity inherent in our cohort, which included both growing (adolescent) and non-growing (adult) patients. Skeletal maturity significantly influences condylar remodeling capacity, soft tissue adaptation, and long-term surgical outcomes [45]. While we recognize the importance of stratifying patients by developmental status, the current sample size (59 patients, 101 joints) does not provide sufficient statistical power for well-stratified subgroup analyses. Consequently, our findings represent aggregate outcomes across different developmental stages, which may mask distinct adaptation patterns in growing versus non-growing individuals. This heterogeneity should be addressed in future multicenter prospective studies with larger, adequately powered samples.

Additionally, potential confounders—including skeletal morphology (e.g., Angle's classification), occlusal characteristics (e.g., overjet, overbite), duration of anterior disc displacement, and parafunctional habits (e.g., bruxism)—were not controlled for due to the retrospective design and sample size constraints. These factors may influence both baseline disease severity and postoperative outcomes [15, 43]. The relatively small sample size precluded comprehensive multivariable modeling with multiple covariates. Therefore, our findings should be interpreted with caution, and causal inferences cannot be drawn.

### MRI Measurements and clinical correlates

We observed a dissociation between anatomical reduction and functional restoration: although disc-condyle mobility improved significantly postoperatively, it remained lower than in group N, suggesting incomplete biomechanical rehabilitation. The correlation between MRI-measured disc-condyle mobility and MMO, while statistically significant, was modest, underscoring that imaging findings alone cannot fully capture functional outcomes. This may be due to the complexity of TMJ kinematics, which involves not only disc position but also muscle coordination, joint loading, and patient adaptation [3, 26, 27]. Comprehensive clinical assessment, including pain scores and quality of life measures, remains essential for evaluating surgical success.

### Clinical Implications and study strengths

Despite its limitations, this study provides clinically actionable insights. The identification of an optimal surgical target range (0° to −60°) offers surgeons a quantitative guide for intraoperative decision-making. Achieving this range may enhance postoperative stability and coordinated disc-condylar movement, potentially reducing the need for prolonged occlusal splint therapy or orthodontic intervention [44]. Furthermore, the use of biplanar MRI allowed us to quantify persistent coronal displacement, highlighting the need for three-dimensional assessment in future studies.

This study has several strengths: (1) systematic evaluation of 101 joints with complete 6-month follow-up; (2) quantitative biplanar (sagittal + coronal) MRI analysis, providing more comprehensive assessment than previous studies [29, 30, 36]; (3) longitudinal tracking of disc-condyle angle and mobility, offering a temporal and kinematic perspective; (4) proposal of a data-driven optimal angle range for surgical targeting, moving beyond binary “reduced vs. non-reduced” assessments common in the literature.

### Limitations

This study has several limitations that should be acknowledged. First, the retrospective design precluded prospective sample size estimation and introduced potential selection and information biases. Although consecutive enrollment minimized selection bias, the relatively small sample size (59 patients, 101 joints) limited statistical power for subgroup analyses and multivariable adjustments.

Second, significant biological heterogeneity existed in our cohort, which included both growing (adolescent) and non-growing (adult) patients. Skeletal maturity influences condylar remodeling capacity and soft tissue adaptation, potentially affecting long-term outcomes. The current sample size was insufficient for well-stratified subgroup analyses, and our aggregate findings may mask distinct adaptation patterns across developmental stages.

Third, the control group consisted of symptomatic non-ADD patients rather than truly asymptomatic volunteers. While this provides a clinically relevant reference, it does not represent a true normative baseline. Future studies should include an asymptomatic control group to establish physiological benchmarks.

Fourth, potential confounders—including skeletal morphology (e.g., Angle's classification), occlusal characteristics (e.g., overjet, overbite), duration of anterior disc displacement, and parafunctional habits (e.g., bruxism)—were not controlled for due to the retrospective nature and sample size constraints. These factors may influence both baseline disease severity and postoperative outcomes, introducing residual confounding.

Fifth, although MRI measurements demonstrated excellent intra-observer reliability (ICC > 0.90), inter-observer validation was not performed, which may limit generalizability. Additionally, the correlation between MRI-measured disc-condyle mobility and clinical functional parameters (e.g., MMO) was modest, underscoring that imaging findings alone do not fully capture functional recovery.

Sixth, the follow-up period of 6 months is relatively short; longer-term studies (> 12 months) are needed to assess the durability of surgical outcomes and potential late relapse or adaptation.

Seventh, we did not systematically collect semi-quantitative clinical data (e.g., pain scores, quality of life measures), which would have enriched the interpretation of functional improvements.

Despite these limitations, this study provides valuable quantitative data on postoperative disc-condyle dynamics and establishes an evidence-based angular target for surgical planning. Future prospective multicenter studies with larger, well-characterized cohorts, longer follow-up, and comprehensive confounding control are warranted to validate and extend these findings.

## Conclusion

In patients with ADDWoR, Bi-ADRO significantly improves sagittal disc position and joint mobility within 6 months postoperatively, although coronal disc displacement may persist. The degree of surgical overcorrection, quantified by the closed-mouth disc-condyle angle, correlates with postoperative stability and coordinated disc-condylar movement. A surgical target angle of 0° to −60° is associated with favorable short-term outcomes. These findings provide a quantitative framework for surgical planning and postoperative assessment. Prospective studies with longer follow-up, larger sample sizes, and adjustment for confounding variables are needed to validate these observations and establish long-term efficacy.

## Data Availability

No datasets were generated or analysed during the current study.

## Abbreviations

Bi-ADRO: Bilateral arthroscopic disc repositioning operation
MRI: Magnetic resonance imaging
ADD: Anterior disc displacement
TMJ: Temporomandibular joint
TMD: Temporomandibular disorder
